# YouTube in medical education: a student's perspective

**DOI:** 10.3402/meo.v20.29507

**Published:** 2015-09-16

**Authors:** Riham Rabee, Muhammad Najim, Yusuf Sherwani, Maroof Ahmed, Muhammad Ashraf, Osama Al-Jibury, Nuha Rabee, Rula Najim, Aaniya Ahmed

**Affiliations:** 1 Medical Department at Imperial College, London University, South Kensington, London, UK; 2 Newcastle High School for Girls, Chapman House, Sandyford Park, Newcastle upon Tyne, London, UK

YouTube, a decade on from its inception, exceeding 1 billion users (1), has become the largest video-sharing platform on the Internet. With more than 300 hours of video uploaded every minute (1), generating millions of daily views, YouTube has succeeded in becoming a podium for all video sectors.

With this expansion, a new division has emerged over the years: Education. YouTube is an extremely practical teaching tool, with over half (1) of viewings on handheld mobile devices, not limited by time or place, unlike books, lectures, and tutorials. YouTube has been used to teach preschool learners through to graduate level and beyond.

Interestingly, 46% of all UK YouTube users are 18–24 year olds (2), which only begs the question: Why have medical institutes not promoted YouTube as a rich educational resource?

Azer et al. (3) compared the content of textbooks, eMedicine articles, and YouTube on cardiovascular mechanism, and found that YouTube excelled not only on the user interface front but also in terms of content and integrating of information across a molecular and clinical level. He found that YouTube offered up-to-date and digestible educational resources to medical students, with a bonus feature of interactivity between users via promotion of user comments and feedback.

Throughout my years at medical school, YouTube was vital in my pre-clinical years, in particular laying down my foundations of anatomy, and later becoming my main source of information for OSCE and practical skills in my clinical years. These collections of videos have created a snowball effect with new channels being created purely for teaching OSCE examinations.

With YouTube videos such as Dr Najeeb's Lectures reaching over 17 million views (4), gaining cult status across medical campuses in the United Kingdom, YouTube has certainly become a vital part of medical school life.

However, despite all its benefits, there are problems that need to be addressed. Studies have shown that often the videos are inaccurate with no regulation of content (5–7). There has also been a recent overcrowding of YouTube, with too much selection, often leaving students confused as to where to look for knowledge.

I therefore believe that there is a necessity for increased recognition by medical institutes of these YouTube videos. The endorsement of these medical videos should be at an institutional level whereby universities not only encourage the use of these resources, but also get involved in making these videos to ensure a more regulated, accurate, and tailored approach for the benefit of their medical students.

Riham Rabee Medical Department at Imperial College London University, South Kensington London, UK Email: Riham.rabee10@imperial.ac.uk Muhammad Najim Medical Department at Imperial College London University, South Kensington London, UK Yusuf Sherwani Medical Department at Imperial College London University, South Kensington London, UK Maroof Ahmed Medical Department at Imperial College London University, South Kensington London, UK Muhammad Ashraf Medical Department at Imperial College London University, South Kensington London, UK Osama Al-Jibury Medical Department at Imperial College London University, South Kensington London, UK Nuha Rabee Newcastle High School for Girls Chapman House, Sandyford Park Newcastle upon Tyne London, UK Rula Najim Medical Department at Imperial College London University, South Kensington London, UK Aaniya Ahmed Medical Department at Imperial College London University, South Kensington London, UK
